# Mechanisms and treatment of late-life depression

**DOI:** 10.1038/s41398-019-0514-6

**Published:** 2019-08-05

**Authors:** George S. Alexopoulos

**Affiliations:** Weill Cornell Institute of Geriatric Psychiatry, 21 Bloomingdale Road, White Plains, NY 10605 USA

**Keywords:** Depression, Psychiatric disorders

## Abstract

Depression predisposes to medical illnesses and advances biological aging indicated by shorter telomere length, accelerated brain aging and advanced epigenetic aging. Medical illnesses also increase the risk of late-life depression. The reciprocal relationships of depression with aging-related and disease-related processes have generated pathogenetic hypotheses and provided treatment targets. Targeting risk factors of vascular disease in mid-life is a logical approach in prevention of vascular depression. The depression-executive dysfunction and the vascular depression syndromes have clinical presentations and neuroimaging findings consistent with frontostriatal abnormalities. Dopamine D_2/3_ agonists are effective in depression of Parkinson’s disease and their efficacy needs to be assessed in these two syndromes. Computerized cognitive remediation targeting functions of the cognitive control network may improve both executive functions and depressive symptoms of late-life major depression. Significant progress has been made in neurostimulation treatments in depressed younger adults. TMS targeting deep structures responsible for mood regulation is well tolerated by older adults and its efficacy in syndromes of late-life depression needs to be studied. Efficacious psychotherapies for late-life depression exist, but are underutilized in part because of their complexity. Streamlined, stepped psychotherapies targeting behaviors assumed to result from dysfunction of brain networks implicated in late-life depression can be easy to learn and have potential for dissemination. However, their effectiveness needs further investigation. Depression increases the risk of dementing disorders. Antidepressants are rather ineffective in treating depression of demented patients, but long-term use of antidepressants may reduce the risk of dementia. However, confirmation studies are needed.

## Introduction

**Fig. 1:**
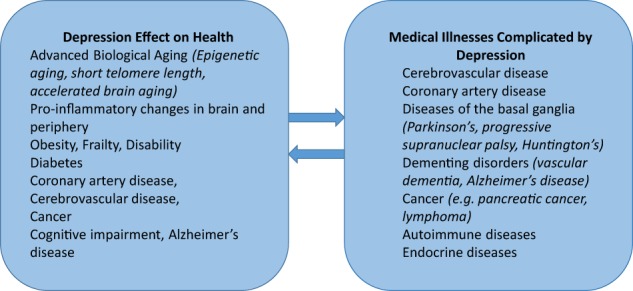
Reciprocal relationship of depression and medical health

Depression advances biological aging evidenced by shorter telomere length, accelerated brain aging and advanced epigenetic aging^1^. Depression increases the risk of obesity, frailty, diabetes, cognitive impairment, and mortality^2,3^. A body of literature links depression with cardiac, cerebrovascular, and peripheral arterial diseases^4^. Depressed individuals have 45% (95% CI: 1.29–1.63) higher risk for stroke than non-depressed individuals and 25% (95% CI: 1.11–1.40) higher risk for stroke-related mortality^5^. Medical illnesses, including cardiovascular and cerebrovascular diseases, are often accompanied by depression^6,7^. Taken together, these observations indicate that depression predisposes to a variety of medical illnesses but also medical illnesses increase the risk of late-life depression (LLD) (Fig. 1). The reciprocal relationships of depression with aging-related and disease-related processes have generated hypotheses on the etiopathogenesis of LLD syndromes and provided targets for treatment development. This review focuses on this work and its implications for novel therapeutics.

## Mechanisms of late-life depression

**Fig. 2:**
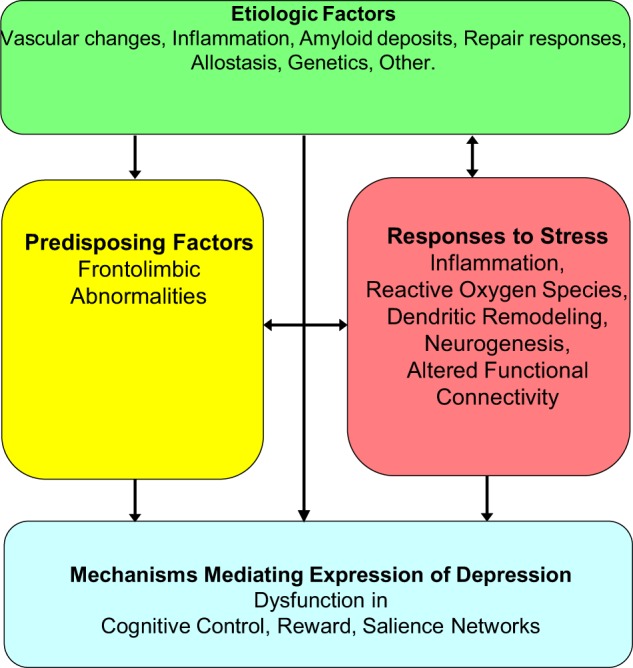
Working model of late-life depression

A working model of LLD postulates that the depressive syndrome represents the clinical expression of dysfunction in reward, salience and cognitive control networks^8–11^ (Fig. 2). The degree of dysfunction in these networks may determine the intensity of symptoms related to mood, cognition, and/or motoric behavior and account for the heterogeneous clinical presentations of the late-life depressive syndrome. Abnormalities in overlapping and/or distinct networks within the frontolimbic system may serve as predisposing factors, facilitating the functional abnormalities mediating the expression of depression, and promoting chronicity and relapse^12^. Genetic factors, aging, and disease-related processes (e.g., inflammation, vascular disease, amyloid accumulation)^7,13–15^ may serve as etiological factors by either directly promoting dysfunction in reward, salience, and cognitive control networks and/or by compromising frontolimbic networks predisposing to depression. Many of the etiological factors start in mid-life, e.g., hypertension, diabetes, obesity, vascular and hormonal changes, amyloid deposition, inflammatory responses, changes in neuroplasticity and synaptogenesis. Late-life and mid-life is often associated with medical and psychosocial problems at the individual (pain, unemployment, elder mistreatment, divorce/widowhood, poverty, social isolation) but also at the community level (rising costs/fixed income, limited access to health care, crime). These stressors may lead to inflammatory responses, increased reactive oxygen species, suppressed neurogenesis, and promote apical dendritic atrophy in the medial prefrontal cortex, and altered functional connectivity (FC)^16^. Stress responses may, then, lead to depression directly by triggering dysfunction in the reward, salience, and cognitive control networks, by promoting frontolimbic abnormalities predisposing to depression, or by increasing aging or disease related processes serving as etiological factors of depression directly (e.g., through allostasis^17^) or indirectly through neglect of health. This model has served to organize testable hypotheses of relationships among etiological, predisposing, and stress related factors and mechanisms mediating the behavioral expressions of LLD and course of illness.

The syndromes described below are based on hypotheses related to the working model of LLD. Although described separately for simplicity, some of their mechanisms overlap and additional mechanisms may even be at play. For example, the depression-executive dysfunction syndrome is the clinical expression of frontostriatal dysfunction often contributed by cerebrovascular dysfunction, abnormal inflammatory responses, and perhaps amyloid deposition. By the same token, the vascular depression syndrome may present with symptoms originating from frontostriatal dysfunction caused by vascular lesions disconnecting networks related to mood regulation and executive functions, as well as reduced cerebral blood flow and inflammatory responses. Data-driven multidimensional approaches began to identify mediators of cognitive impairment of patients with LLD. Machine learning of proteomic data and measures of structural brain abnormalities and brain amyloid-β (Aβ) PET scans showed that cognitive impairment in LLD is related to greater cerebrovascular disease along with abnormalities in immune-inflammatory control, cell survival, intracellular signaling, protein and lipid homeostasis, and clotting processes^18^. Finally, a senescence associated phenotype consisting of 22 proteins was found elevated in LLD and associated with medical and cognitive burden^19^ indicating another source of vulnerability.

### The depression-executive dysfunction syndrome hypothesis

A depression executive dysfunction (DED) syndrome has been described in older adults with distinct clinical presentation and poor response to antidepressants^20^. Approximately 30% of depressed older adults have abnormal performance in tests of verbal fluency, response inhibition, novel problem solving, cognitive flexibility, working memory, and/or ideomotor planning^11,21^ (Table 1). The depression profile of DED is characterized by anhedonia, psychomotor retardation, pronounced disability, lack of insight, and suspiciousness but less prominent depressive ideation and a mild vegetative syndrome^22–24^. This presentation is consistent with disruption of frontal-subcortical networks. Depression frequently develops in disorders of subcortical structures, including vascular dementia, Parkinson’s disease, Huntington’s disease, supranuclear palsy and basal ganglia calcification, and stroke of the caudate head. White matter hyperintensities (WMH) are common in geriatric depression and often located in subcortical structures and their frontal projections^25^. Diffusion tensor imaging studies of LLD identified microstructural abnormalities in white matter tracts that connect the prefrontal cortex with subcortical and posterior cortical regions, which have been linked to executive dysfunction^26,27^. Low metabolic activity and resting FC have been observed in the dorsal anterior cingulate cortex (dACC) and the dorsolateral prefrontal cortex (DLPFC) during depressive episodes in older adults^8,28^. Tasks challenging the cognitive control network resulted in hypoactivation of the DLPFC in LLD and diminished FC between the DLPFC and the dACC^28^. Hypoactivation of the DLPFC resolved after SSRI treatment but decreased task-based FC persisted^28^.

**Table 1:** Findings related to the depression executive dysfunction syndrome hypothesis

Presentation
• Anhedonia, psychomotor retardation, pronounced disability, lack of insight, and suspiciousness but less prominent depressive ideation and a mild vegetative syndrome
• Impaired verbal fluency, response inhibition, novel problem solving, cognitive flexibility, working memory, and/or ideomotor planning
Neuroimaging
• White matter hyperintensities and microstructural abnormalities in white matter tracts connecting the prefrontal cortex with subcortical and posterior cortical regions
• Hypoactivation of DLPFC during tasks challenging the cognitive control network and diminished functional connectivity between the DLPFC and the dACC
• Diminished functional connectivity between the DLPFC and the dACC persists after antidepressant treatment
Treatment response
• Poor response to antidepressants and early relapse and recurrence
• Low white matter integrity in distributed networks tracts supporting executive functions was associated with poor response of late-life major depression to a serotonin reuptake inhibitor
• Low activation in the DLPFC and other brain regions during the Wisconsin Card Sorting Test of executive functions predicted less favorable response to cognitive behavioral therapy in depressed, older adults

Apathy is common in LLD and associated with executive dysfunction, disability, and poor antidepressant response^29^. The impairment in executive functions tests of DED patients may in part be due to the motivational disturbance of apathy. Alternatively, apathetic DED, may be a subtype of DED in which a shared neurobiological dysfunction leads to depressed mood, apathy, and executive dysfunction. Apathy is associated with reduction in white matter integrity in the anterior cingulum, fornix, and uncinate fasciculus^30^. Older apathetic patients with major depression had lower resting FC of the nucleus accumbens with the amygdala, caudate, putamen, globus pallidus, and thalamus and increased FC with the dorsomedial prefrontal cortex, the superior frontal cortex, and the insula than non-apathetic patients^31^. Further, apathetic depressed patients had lower resting FC of the dACC with dorsolateral and ventrolateral prefrontal cortices and higher FC with the insula and the orbitofrontal cortex than non-apathetic depressed patients^31^. Also, depressed elderly patients had decreased intrinsic resting FC of the salience network and an altered pattern of salience network FC to the right DLPFC node of the cognitive control network when compared to elderly non-apathetic depressed and to normal, elderly subjects^9^. These observations suggest that abnormal FC of the reward, salience and cognitive control networks underlies apathy of LLD.

Executive dysfunction predicts poor response of LLD to antidepressants and early relapse and recurrence^32–38^. Subcortical WMHs are common in LLD and have been associated with both executive dysfunction and non-remission of LLD^39^. Diffusion tensor imaging showed that lower white matter integrity in distributed networks tracts (dorsal and rostral ACC, DLPFC, hippocampus, posterior cingulate, insula, neostriatum, and the midbrain, as well as select temporal and parietal regions) was associated with poor response of late-life major depression to a serotonin reuptake inhibitor^26^. Low resting FC within the networks supporting executive functions, but not within the default mode network (DMN), predicted persistence of depressive symptoms and signs, apathy, and dysexecutive behavior after treatment with escitalopram^31^. Similarly, lower activation in the DLPFC and other brain regions during in-scanner performance of the Wisconsin Card Sorting Test of executive functions predicted less favorable response to cognitive behavioral therapy in depressed, older adults^40^. The poor response of DED to antidepressants and the evolving understanding of its pathogenesis may guide the development of targeted interventions.

### The vascular depression hypothesis

The ‘vascular depression’ hypothesis postulates that cerebrovascular disease may predispose, precipitate, or perpetuate some geriatric depressive syndromes^13,41^. This hypothesis was based on the presence of cerebrovascular risk factors in many patients with LLD, the comorbidity of LLD with cerebrovascular lesions, and the frequent development of depression after stroke.

A clinical definition regards cerebrovascular risk factors or cerebrovascular disease as one of the cardinal features of vascular depression (Table 2). Cerebrovascular risk factors are associated with WMH in healthy young adults^42^. Elevated systolic blood pressure has been associated with brain infarcts, gross infarcts, and microinfarcts^43^. Vascular risk factors lead to vascular wall hypertrophy, increased intima media thickness, reduced arterial distensibility, and endothelial cell dysfunction^44^. Such vascular changes have been associated with poor response to antidepressants^45^. MRI stigmata of cerebral small vessel disease, (i.e., WMHs, lacunes, microbleeds, perivascular spaces, and cerebral atrophy) are associated with depression and incident stroke^46^. The Cardiovascular Health Study showed that persistence of depressive symptoms was associated with small basal ganglia lesions and large cerebral cortical white-matter lesions while worsening of depression severity was associated with subcortical white-matter lesions^47^. Greater arterial stiffness (carotid-femoral pulse wave velocity) was associated with depressive symptoms; this relationship was partly accounted by white WMH volume and subcortical infarcts^48^. Markers of progression of cerebral small vessel disease (WMH volume, subcortical infarcts, cerebral microbleeds, Virchow-Robin spaces, and total brain volume) over time were associated with new depressive symptoms in community elders^49^. Carotid plaque presence was associated with higher severity of depressive symptoms at a 10-year follow-up in men^50^.

**Table 2:** Findings related to vascular depression hypothesis

Clinical picture
• Onset of depression in late-life or worsening of the course of early-onset depression after the onset of vascular disease
• Cerebrovascular risk factors, arterial stiffness (carotid-femoral pulse wave velocity), carotid plaques
• Neuropsychological impairment, including executive dysfunction, depending on the location and extent of cerebrovascular lesions
• Depression usually characterized by retardation, anhedonia, lack of insight into their illness, and disability, but less feelings of guilt
• Poor or slow response to antidepressants
Neuroimaging
• Hyperintensities in subcortical gray matter, deep white matter, or periventricular areas
• Low cerebral blood flow (CBF) in the precuneus, cuneus, in fronto-cingulate-striatal areas, temporal, occipital, and parietal lobes and high CBF in frontal and temporal cortices and the cingulate gyrus by pulsed arterial spin labeling
• Low resting functional connectivity of the subgenual ACC and high connectivity of the DMPFC
• High activation of the subgenual cingulate in response to an affective-reactivity task suggesting limbic hyperactivation
• Low activation of DLPFC during a continuous performance task and low connectivity with task-relevant brain regions including middle frontal gyrus, and supramarginal gyrus
Other findings
• Circulating markers of endothelial dysfunction and flow mediated vascular dilatation
• Disruption of immune functions
• Increased hypothalamic-pituitary-adrenal axis
Negative neuropathology findings
• Lacunes and microvascular ischemic lesions were not related to occurrence of late-onset depression
• Gross or microscopic infarcts were not associated with severity of depressive symptoms or change of depressive symptoms overtime

A second cardinal feature of the clinical definition of vascular depression is either onset in late-life or worsening of the course of early-onset depression after the onset of vascular disease. Early onset does not preclude the diagnosis of vascular depression since history of depression increases the risk of vascular disease and stroke^41,51^ and may promote inflammation^52,53^ or epigenetic changes of genes related to vascular integrity^14,54,55^, suggesting that depression has a bidirectional relationship with vascular diseases.

Neurological signs and/or neuropsychological findings, usually executive dysfunction, are found in most patients with vascular depression depending on the location and extent of lesions. Late onset and absence of family history of mood disorders are expected in most cases but family history of mood disorders does not preclude vascular depression, since family history of mood disorders was shown to predispose to post-stroke depression^56^. Patients with vascular depression often present with retardation, anhedonia, lack of insight into their illness, and disability and are less likely to report feelings of guilt^23,57^.

An MRI based definition of vascular depression requires presence of hyperintensities in the subcortical gray matter, deep white matter, or periventricular areas^41,57^. Compromised white matter integrity is associated with LLD and predicts future depressive symptoms^58^. Depression has been associated with greater WMH severity in white matter tracts of the cingulum, uncinate fasciculus, and superior longitudinal fasciculus^59,60^, as well as the frontal^25^ and temporal lobes^61^. Diffusion tensor imaging studies have shown reduced anisotropy in the DLPFC and the uncinate fasciculus of patients with LLD consistent with disruption of frontal and frontal-to-limbic white matter tracts^62^. Depressed older adults were shown to have decreased resting FC in the subgenual anterior cingulate cortex and increased connectivity in the dorsomedial prefrontal cortex and the orbitofrontal cortex;^63^ abnormal FC was correlated with greater WMH volume. High WMH burden in LLD was associated with greater activation of the subgenual cingulate in response to a facial expression affective-reactivity task, suggesting that white matter ischemic changes lead to limbic hyperactivation^64^.

Some neuropathology studies failed to identify a relationship between vascular brain lesions and depression. Neither lacunes nor microvascular ischemic lesions were related to occurrence of late-onset depression^65,66^. Further, gross or microscopic infarcts were not associated with severity of depressive symptoms or change of depressive symptoms overtime^67,68^.

WMH burden is associated with executive dysfunction and reduced activation of brain regions related to executive and psychomotor functions. Executive dysfunction was associated with bilateral WMH in the inferior frontal white matter, temporal-occipital periventricular white matter, and the anterior limb of the internal capsule, as well as scattered clusters in the prefrontal white matter^69^. WMHs were correlated with impairments in goal maintenance during a continuous performance task, but also with reduced activity in DLPFC and reduced connectivity of the DLPFC with task-relevant brain regions including middle frontal gyrus, and supramarginal gyrus^70^. In addition, WMHs were associated with increased activity in the anterior cingulate on a facial expression affective-reactivity task^64^.

A confluence of interacting events may lead to vascular depression. WMHs and microstructural abnormalities may damage fiber tracts including the cingulum, uncinate fasciculus, anterior thalamic radiation, and superior longitudinal fasciculus^59,60,71,72^ and lead to disconnection and dysfunction of networks supporting affective and cognitive functions^73^. LLD patients were shown to have an anterior-posterior gradient in cerebral blood flow (CBF), with lower CBF throughout the frontal lobe but higher CBF in the parietal lobe, temporal lobe, thalamus, and hippocampus^74^. A similar anterior to posterior gradient was observed in the cingulate cortex. Aging related vascular pathology reduces blood flow velocities and decreases vasomotor reactivity^75^, compromising CBF. Large impairment in perfusion and autoregulation may result in WMH and gray matter lesions^76^. Circulating markers of endothelial dysfunction and flow mediated dilatation were correlated with depressive symptoms in a community of elderly population^77^. Pulsed arterial spin labeling showed that relative to healthy controls, remitted late-onset depressed patients had decreased cerebral blood flow in the precuneus and cuneus bilaterally and in the right fronto-cingulate-striatal areas, temporal, occipital, and parietal lobes, but increased CBF in the left frontal and temporal cortices and the cingulate gyrus^78^. A meta-analysis reported that higher levels of the plasma endothelial biomarker soluble intercellular adhesion molecule-1, WMH, cerebral microbleeds, and cerebral micro-infarctions are associated with depression; WMH were associated with incident depression^79^. A recent study reported an association of WMH with tumor necrosis factors alpha (TNF-α) and interferon gamma (INFγ) and macrophage inflammatory protein-1α^80^. Older adults with high homocysteine plasma level had increased risk of depression^81^. Aging related disruption of immune functions contribute to WMH burden and predispose to LLD^82^. Increased hypothalamic-pituitary-adrenal (HPA) axis function during depressed states may influence inflammatory responses. Amyloid deposition in and around cerebral blood vessels may change the integrity of blood–brain barrier and release of inflammatory mediators, which may damage the basal lamina and increase the risk of microhemorrhages^7^. In a transgenic mouse model of Alzheimer’s disease, cortical arterioles had Aβ accumulation, high tortuosity, and narrow caliber and their function was compromised^83^. Inhibition of Aβ oligomerization and fibrillization prevented both structural and functional impairment of the cortical microvasculature. Interactions among the above processes, and processes yet to be identified, may provide targets for prevention or treatment of vascular depression.

### The inflammation hypothesis

The inflammation hypothesis posits that age-related and comorbid disease-related immune deregulation contribute to the etiology of LLD^84^ (Table 3). Aging leads to a pro-inflammatory changes mediated by increased immune responses in the periphery, disruption of the periphery-brain immune communication, and an increased and discordant brain response^85^. Disruption in the periphery-brain immune communication, produces a disproportionate brain inflammatory response to peripheral immune stimulation, promoting a chronic proinflammatory state with increased activated and primed microglia, continuous production of pro-inflammatory cytokines IL-1β, IL-6, and TNF-α, and decreases in anti-inflammatory molecules^86^. Persistent activation of microglia leads to inefficient clearance of neurotoxic molecules, neuron loss, and reduction of neurogenesis^87^.

**Table 3:** Findings relevant to the inflammation hypothesis of late-life depression

Mechanisms
• Aging increases immune responses in the periphery, disrupts the periphery-brain immune communication, and increases activated and primed microglia leading to production of pro-inflammatory cytokines and also in reduction of anti-inflammatory molecules
• Persistent activation of microglia leads to inefficient clearance of neurotoxic molecules, neuron loss, and reduction of neurogenesis
• Cytokines induce indoleamine 2,3-dioxygenase, an enzyme that reduces serotonin production
• Cytokines dysregulate the glutamate system, promote excitotoxicity and decrease production of neurotrophic factors that promote neuroplasticity, and neurogenesis
• Cytokines increase oxidative stress, which damages glial cells in the prefrontal cortex and the amygdala
• Inflammation may disrupt glucocorticoid receptor function and increase inflammatory responses that fuel depressive symptoms
• Inflammatory responses to immune challenge influence the function of emotional networks
• Peripheral inflammatory markers are elevated in late-life depression and their levels are associated with severity of depression and with cognitive symptoms of depression
• Pro-inflammatory changes have been documented in diseases and health risk factors predisposing to late-life depression including cardiovascular disease, high body mass index, smoking, and chronic stress
• Long duration of untreated major depressive disorder predicts activation of microglia
Treatment
• Antidepressants reduce peripheral markers of inflammation
• In depressed patients with low C-reactive protein (CRP), escitalopram was more efficacious than nortriptyline, while nortriptyline was more efficacious in patients with higher CRP levels
• The TNF-α antagonist infliximab reduced symptoms of major depression in individuals with baseline high-sensitivity CRP (hs-CRP), while individuals with lower hs-CRP concentrations did better with placebo
• Nonsteroidal anti-inflammatory drugs (NSAIDs), omega-3 fatty acids, and cytokine antagonists may have antidepressant properties in individuals with major depression and high inflammatory biomarkers

Cytokines induce indoleamine 2,3-dioxygenase, an enzyme that reduces serotonin production^88^. They also dysregulate the glutamate system, promote excitotoxicity and decrease production of neurotrophic factors, neuroplasticity, and neurogenesis. In major depression, plasma C-reactive protein was correlated with concentrations of glutamate in the left basal ganglia^89^. Administration of the pro-inflammatory interferon alpha (IFN-α) increased glutamate in the basal ganglia of non-depressed subjects with hepatitis C;^90,91^ changes in glutamate concentrations were in turn associated with anhedonia and psychomotor slowing. Cytokines contribute to oxidative stress, which damages glial cells in the prefrontal cortex and the amygdala^92^. Inflammation may cause resistance to glucocorticoids in immunocytes and their cellular targets^93^, disrupt glucocorticoid receptor function and increase inflammatory responses that further fuel depressive symptoms.

Inflammatory changes in the brain have been associated with depression. A PET study used F-FEPPA ligand to measure translocator protein total distribution volume (TSPO V_T_), a marker of microglial activation^94^. Duration of untreated major depressive disorder was a strong predictor of TSPO V_T_, as were total illness duration, and duration of antidepressant exposure. The combination of these predictors accounted for about 50% of variance in TSPO V_T_ in the prefrontal cortex, anterior cingulate cortex, and insula^94^. Increased cell adhesion molecule expression has been found in the DLPFC in LLD, an inflammatory response associated with ischemia^95^.

Inflammatory responses to immune challenge influence the function of emotional networks. A SNP encoding IL-1β has been associated with both reduced activity of the anterior cingulate and the amygdala in response to emotional probes and with poor response of major depression to antidepressants^96^. Patients treated with the cytokine INF-α exhibited greater dorsal anterior cingulate activation than controls;^97^ dysfunction of the anterior cingulate has been documented in geriatric depression^98^. Enhanced activation of the subgenual anterior cingulate cortex during emotional face processing and reduced FC of the subgenual anterior cingulate with the amygdala, medial prefrontal cortex and nucleus accumbens is modulated by IL-6^99^.

Peripheral inflammatory markers are elevated in LLD and their levels are associated with severity of depression^100^ and with cognitive symptoms of depression^101^. A meta-analysis showed that peripheral levels of interleukin‐6 (IL‐6), tumor necrosis factor TNF-α, IL‐10, soluble IL‐2 receptor, C–C chemokine ligand 2, IL‐13, IL‐18, IL‐12, IL‐1 receptor antagonist, and soluble TNF receptor 2 were elevated and interferon‐gamma levels were lower in individuals with major depression compared to controls^102^. Elevated IL-6 is associated with increased suicide risk, with the highest levels of IL-6 correlating with the most violent suicide attempts^103^. High IL-1ra levels have been found in older adults with depressive symptoms and have been a risk factor for developing depressive symptoms during a 6 year follow-up^104^. Antidepressant treatment significantly decreased peripheral levels of IL-6, TNF-α, IL-10, and CCL-2^105^.

Pro-inflammatory changes have been documented in medical illnesses and health risk factors predisposing to LLD. Increased circulating inflammatory cytokines have been found in cardiovascular disease^106^. High body mass index and smoking have been associated with increased inflammatory markers in major depression^102^. Chronic stress, a precipitant of depression, exacerbates age-related increases in inflammatory responses and increases circulating IL-1β and IL-6 and cognitive impairment in elderly patients^107^. Although peripheral cytokines do not cross the blood–brain barrier, they send signals via molecular, cellular, and neural routes, which ultimately enhance brain inflammation^15,108^. Aging may exacerbate the effects of stress in the brain, leading to behavioral and cognitive changes similar to those of depressive syndromes.

### Is amyloid and Tau accumulation one of the mechanisms of LLD?

Several studies suggest that amyloid beta (Aβ) accumulation may predispose to LLD^109^. In cognitively unimpaired older adults, increased amyloid burden in the precuneus/posterior cingulate cortex were associated with depressive symptoms^110^. In community-dwelling, cognitively unimpaired elderly individuals, Aβ burden was associated with increasing anxious-depressive symptoms during a 1–5 year follow-up (mean = 3.8 years)^111^. Patients with a lifetime history of depression had amyloid accumulation in brain regions related to mood regulation^112^. Depression is associated with a high conversion rate of amnestic mild cognitive impairment (aMCI) to Alzheimer’s dementia^113^. Patients with aMCI and history of major depression had higher Aβ deposition, mainly in the frontal cortex, compared to patients with aMCI without history of major depression^114^. Alzheimer’s patients with history of depression had more amyloid plaques in the hippocampus than Alzheimer’s patients without depression^115^. Individuals with LLD had lower plasma Aβ_42_ levels and a higher plasma Aβ_42_/Aβ_40_ ratio than did those without depression in the absence of cardiovascular disease and antidepressant use; high plasma Aβ_42_/Aβ_40_ increases the risk of Alzheimer’s disease^116^.

A single dose of citalopram decreased Aβ in the brain’s interstitial fluid in a dose-dependent manner in aged, transgenic (APP/PSI), plaque bearing, AD mice^117^. Chronic administration of citalopram arrested the growth of preexisting plaques and the development of new plaques by 78%^117^. In healthy individuals, acute administration of citalopram 60 mg slowed the production of Aβ in the CSF by 37% compared to placebo^117^. Community volunteers treated with antidepressants over a period of 5 years (mean: 34.5 months) had significantly lower amyloid load in brain PET scans than those who had never received antidepressants^118^. The length of antidepressant treatment prior to scanning correlated with lower plaque load. Finally, depression increases the risk of conversion of MCI to Alzheimer’s dementia^113^ and long-term treatment with antidepressants delays the conversion of mild cognitive impairment to Alzheimer’s dementia^119^.

Despite the above findings, several studies failed to identify a relationship between Alzheimer’s pathology and LLD. A neuroimaging study found no differences in cortical Aβ uptake or in the proportion of amyloid-positive subjects between depressed older patients and healthy controls^120^. Non-demented patients with prior depressive episodes had cortical Aβ levels indistinguishable from healthy controls^121^. An early neuropathology study reported no significant differences in plaque or tangle counts between subjects who were cognitively impaired and those who were unimpaired during their depressive illness^122^. A more recent study found no differences in neuritic pathology or neuronal density between the subjects with primary major depression and nondepressed comparison subjects^123^. Subjects with Alzheimer’s disease had fewer serotonergic neurons and more neuritic pathology, compared to depressed subjects and healthy controls but there were no differences between depressed and non-depressed Alzheimer’s disease subjects on these measures. Another neuropathology study found no significant association between depressive symptoms cognitive status, neuritic plaque, and neurofibrillary tangle scores or their interactions^124^. Finally, there were no differences between LLD patients and healthy controls in CSF total and phosphorylated tau^125^. Discrepancies in the studies summarized above make it unclear whether and what aspects of neurobiological changes of Alzheimer’s disease are related to LLD.

## Treatment

Old age is a risk factor for a poor course of major depression, which could not be explained by a range of risk factors^126^. Nonetheless, several treatment options exist (Table 4).

**Table 4:** Evidence-based and novel therapies for late-life depression

Evidence-based therapies
Antidepressants
• Late-life depression responds less well to antidepressants and has a higher relapse rate than the depression of younger adults
• Lithium, aripiprazole, and methylphenidate are efficacious augmentations of antidepressants
• Antidepressants may improve depression of most medical illnesses but it is unclear if they improve the outcomes of medical illnesses
• Antidepressants are generally ineffective in depression of dementia
• Antidepressants may reduce brain amyloid load and long-term treatment with antidepressants may delay the conversion of mild cognitive impairment to Alzheimer’s dementia
Electroconvulsive therapy (ECT)
• Brief pulse right unilateral ECT may be slightly more efficacious than ultra-brief pulse unilateral ECT, but may lead to greater cognitive side effects
• Addition of ECT to continuation pharmacotherapy may reduce relapse rate in antidepressant resistant depression
Psychotherapies
• Problem solving therapy, cognitive behavioral therapy, and interpersonal therapy are effective in late-life depression
• Problem solving therapy is efficacious in depression with executive dysfunction
• Evidence based psychotherapies are rarely used correctly in the community
Novel therapies
Neurobiology-based psychotherapy
• ENGAGE, a stepped therapy for late-life depression, targets behavioral domains grounded on neurobiological constructs using behavioral techniques selected for their simplicity and efficacy
• A proof of concept study showed that ENGAGE is non-inferior to PST and engages its behavioral target
Depression-executive dysfunction syndrome (DED)
• Dopamine receptor D2/D3 agonists may improve depressive symptoms in patients with Parkinson’s disease and in idiopathic depression but definitive studies in DED are lacking
• Computerized cognitive remediation targeting executive functions had similar efficacy with historical controls treated with escitalopram in a preliminary study
Vascular depression
• Addressing modifiable risk factors since early mid-life may reduce the risk of vascular depression
• Angiotensin receptor blockers and some calcium channel blockers can improve cerebral hemodynamics but high quality efficacy studies are lacking in vascular depression
Inflammation hypothesis
• Anti-inflammatory agents and cytokine inhibitors may have antidepressant properties in depressed patients with increased inflammatory markers but confirmation studies are needed

### Antidepressants

Antidepressants are more efficacious than placebo in LLD^127^. In late-life major depression, the response rate to antidepressants is lower compared to depression in younger patients but the placebo response rate is similar^128^. The number of patients with late-life major depression needed to treat (NNT) with antidepressants in order to achieve one more remission compared to placebo was 14.4 (95% CI 8.3–50) and 6.7 (95% CI 4.8–10) in order to achieve one more response^129,130^. Augmentation of antidepressants with either lithium^131^ or with the aripiprazole^132^ have been found effective in late-life major depression unresponsive to an antidepressant. Combination of citalopram and methylphenidate may improve mood and well-being and lead to a higher remission rate of LLD compared to either drug alone^133^. Donepezil added to the maintenance antidepressant therapy of LLD that led to temporary positive effects of donepezil on cognitive function, marginal improvement of cognitive instrumental activities of daily living, and, in those with MCI, a lower rate of conversion to dementia over 2 years^134^. However, another study failed to confirm the benefit of donepezil in reducing conversion to dementia^135^. Donepezil increased the risk of recurrence especially in cognitively impaired LLD patients^134^.

The relapse and recurrence rate of LLD is higher than depression of younger adults^136^. Antidepressants, psychotherapy, or a combination reduce the relapse and recurrence rates of late-life major depression^137–139^. Continuation treatment with antidepressants in LLD has similar efficacy with that in younger adults^140^. However, even with antidepressant treatment over half of remitted LLD patients experienced recurrence, mostly within 2 years^141^. High number of previous episodes, severity and length of the last episode, residual depressive symptoms, length of well intervals, adverse effects of antidepressants, medical burden, disability, and patient preferences may be taken into consideration in determining the duration of maintenance therapy^142,143^.

#### Medical burden

Antidepressants are effective in reducing depression of most but not all medical illnesses, but it is unclear whether treatment of depression improves the outcomes of medical conditions. In patients with major depression and non-dialysis-dependent chronic kidney disease, sertraline did not significantly improve depressive symptoms compared with placebo over 12 weeks^144^. A depression treatment program improved depression and quality of life in cancer patients, but did not prolong survival^145^. Antidepressants reduce depressive symptoms in patients with acute coronary syndrome^146^ but most studies found no benefit in cardiac outcomes^147–149^. An exception is a recent study, which showed that among depressed patients with recent acute coronary syndrome, 24-week treatment with escitalopram compared with placebo resulted in a lower risk for a composite measure consisting of all-cause mortality, myocardial infarction, and percutaneous coronary intervention after a median of 8.1 years^150^.

#### Dementia

Antidepressants are generally ineffective in depression of dementia^151^. They should be considered in patients with history of response to antidepressants in prior episodes of depression or in episodes with a classical presentation of major depression. Recent findings suggest that SSRIs may delay the onset of Alzheimer’s dementia^119^. Lithium inhibits glycogen synthase kinase 3, a key enzyme in the metabolism of amyloid precursor protein and in the phosphorylation of tau protein, and may reduce the risk of Alzheimer’s dementia^152^.

### Somatic therapies

Electroconvulsive therapy (ECT) is the most efficacious treatment for late-life major depression, with a remission rate 60–80%^142,153,154^. ECT is indicated in patients with psychotic depression, inability to respond or tolerate adequate treatment with antidepressants, severe non-psychotic depression, and inability to receive nutrition^155^. ECT may reduce readmissions of psychiatric in-patients with severe mood disorders^156^. ECT is safe and effective in LLD accompanied by Parkinsonism, dementia, and stroke^157^. Brief pulse, right unilateral ECT may be slightly more efficacious than ultrabrief pulse unilateral ECT and require fewer sessions, but may lead to greater cognitive side effects^158^. Nonetheless, right unilateral ultrabrief pulse ECT, combined with venlafaxine led to remission in 61.7% and to response in 70% of patient with late-life major depression and was well tolerated^159^. The mean number of ECT treatments to achieve remission was 7.3 (SD = 3.1). In remitted patients, adding four ECT treatments over 1 month to continuation treatment with venlafaxine and lithium led to better 24-month outcomes than venlafaxine and lithium alone^159^.

### Psychosocial interventions

Psychotherapy is a critical part of the treatment of LLD because intolerance of therapeutic dosages, reduced efficacy, and drug interactions reduce the usefulness of antidepressants. Problem solving therapy, cognitive behavioral therapy, and interpersonal therapy are effective in the treatment of LLD^160^. Meta-analysis of psychotherapy studies in a wide range of late-life depressive syndromes documented that psychotherapy and pharmacotherapy have comparable efficacy^161^.

Problem solving therapy was more effective that supportive therapy in reducing depression and disability in older adults with DED^162,163^, a syndrome with poor response to antidepressants^32–38,164^. Psychosocial interventions may improve the outcomes of depressed, medically compromised older adults. Personalized interventions aiming to increase adherence to treatment for major depression and COPD improved both depressive symptoms and disability^165–167^. Clinical case management reduced the severity of depression and improved disability in low-income, disabled elders suffering from major depression^168^.

### Novel therapies

LLD overall, and DED and vascular depression in particular, respond less well to antidepressants than depression of younger adults. Evidence-based psychotherapies are rarely used correctly in the community. What follows outlines the rationale for novel therapies and preliminary evidence of efficacy (Table 4). Despite the need for new antidepressant treatments and their theoretical appeal, most of the treatments described below are not clinically used because of inadequate empirical evidence, small effect size, cost, complexity of administration, and other factors.

### Neurobiologically based psychotherapy

Despite their efficacy, psychotherapies are rarely used correctly in the community^169^. An important reason is that their complexity exceeds the skills of many community clinicians. It has been proposed that use of neurobiological constructs can guide the selection of targets and lead to a streamlined and personalized psychotherapy that addresses biologically driven, core aspects of LLD. ENGAGE therapy has been the first attempt to streamline psychotherapy for LLD using this approach^170^. ENGAGE is a stepped therapy that targets behavioral domains grounded on neurobiological constructs using behavioral techniques selected for their simplicity and efficacy. Its principal intervention is “reward exposure” intended to target the behavioral expression of positive valence systems’ dysfunction. During treatment, therapists search for barriers to “reward exposure” in three behavioral domains, i.e., “negativity bias” (negative valence system dysfunction), “apathy” (arousal system dysfunction), and “emotional dysregulation” (cognitive control dysfunction), and add strategies targeting these domains when needed. Initial studies suggest that ENGAGE can be taught to community based therapists, is non-inferior to problem solving therapy^171^, and engages its behavioral targets^172,173^.

### Treatments based on the DED hypothesis

#### D2/D3 agonists

The depression-executive dysfunction (DED) syndrome has a slow or poor response to antidepressants^32–38,164^. A dysfunction in the dopamine system has been postulated in DED as this system regulates psychomotor speed, cognitive functions, motivational behavior, and pleasure. SSRIs and tricyclics indirectly increase extracellular dopamine mainly in the prefrontal cortex and it has been suggested that their efficacy is in part related to their ability to influence dopaminergic neurotransmission^174^. The dopamine receptor D2/D3 agonist pramipexole has been shown to improve depressive symptoms, mainly through a direct effect, in patients with Parkinson’s disease^175^. Open‐label studies and one controlled study showed that augmentation of antidepressants with dopamine agonist is efficacious, with the strongest evidence for pramipexole^176–178^. Despite a theoretical rationale for use D2/3 agonists in DED, and its poor response to antidepressants, randomized controlled trials are lacking.

#### Computerized cognitive remediation (CCR)

Computer software have been developed to provide training in tasks dependent on cognitive control functions. Two proof of concept studies showed that such training improved both depressive symptoms and executive functions in late-life major depression^179^ and in the DED syndrome^180^ more than the comparison conditions. CCR is personalized and continuously adapts its level of difficulty to the patients’ aptitude both at baseline and they progress in treatment^179^. Because it is standardized and self-administered, CCR is not subject to therapists’ skill drift. CCR is relatively inexpensive and can be used at the patients’ homes, thus, minimizing barriers to access of care, common in older adults.

### Transcranial magnetic stimulation (TMS)

Advances in TMS^181^ may reach the deep structures implicated in DED. A recent study of deep TMS (H1 coil) delivered over the dorsolateral and ventrolateral prefrontal cortex showed that deep TMS is safe in LLD and led to higher remission rate than sham rTMS (40.0 vs. 14.8%)^182^. Despite its theoretical appeal, no study has been done in DED yet. However, rTMS has been found efficacious in vascular depression, a syndrome often accompanied by executive dysfunction^183^.

### Treatments based on the vascular depression hypothesis

#### Antihypertensive agents

Modifiable cerebrovascular risk factors are associated with WMH in healthy young adults^42^. A recent clinical-pathologic study showed that higher systolic blood pressure (147 vs. 134 mm Hg) increased the odds of having one or more brain infarcts by 46%, a gross infarct by 46%, and microinfarcts by 36%^43^. The 2017 guidelines of the American College of Cardiology and American Heart Association changed the definition of hypertension and define elevated systolic blood pressure as 120–129 mm Hg with diastolic less than 80^184^. Stage I hypertension is now defined as systolic 130–139 and diastolic 80–89^184^.

Dysfunction in hemodynamics, autoregulation, and vessel reactivity is one of the mechanisms of vascular depression. Angiotensin converting enzyme inhibitors (ACEIs) and angiotensin receptor blockers (ARBs) may improve cerebral hemodynamics^185^ and endothelial function^186^, and preserve cognitive function in hypertensive populations^187^. ARBs may be superior to ACEIs^188^, because of their selective binding with the angiotensin receptor type I, since activation of angiotensin type II receptor has protective effects, leading to vasodilation, neuronal differentiation, and axonal regeneration^189^. ARBs improved cerebral autoregulation and attenuated brain injury by hypoperfusion^190^. ARB use has been associated with improvement depression, anxiety, and quality of life^191^. Direct studies are needed to clarify the role of ARBs in the treatment of vascular depression.

Few studies suggest that calcium channel blockers may reduce depressive symptoms. In an early study, nimodipine of vascular depression treated with a variety of antidepressants, addition of nimodipine improved depressive symptoms more than addition of an inactive comparator^192^. In a follow-up study of patients with vascular depression treated with fluoxetine, augmentation with nimodipine reduced depressive symptoms more than addition of placebo^193^. Moreover, a greater proportion of patients treated with fluoxetine–nimodipine (54 vs. 27%) achieved remission, with the number needed to treat (NNT) equal to 4 (95% CI: 2–12). Of those experiencing full remission in the first 61 days, fewer patients on fluoxetine plus nimodipine (3.7%) developed recurrence of major depression as compared to those on fluoxetine alone (35.7%), NNT 3 (95% CI 2–9). Verapamil was found to be associated with reduction of depressive symptoms in hypertensive patients while atenolol was not^194^.

#### Statins

Statins are widely used in the primary and secondary prevention of coronary artery disease, including patients with average cholesterol level. A meta-analysis of seven observational studies suggests that statins have a protective effect against depression^195^. The mechanisms are unclear but reduction of oxidative stress and inflammatory cytokines and improved blood flow may have a neuroprotective effect.

### Treatments based on the inflammation hypothesis

#### Anti-inflammatory agents

LLD, and especially vascular depression, is often accompanied by increased inflammation biomarkers^102^. Inflammatory cytokines may be associated with chronicity of depression^196^. Antidepressants reduce the levels of several peripheral markers of inflammation^105^. However, it remains unclear if reduction in peripheral inflammation is associated with antidepressant treatment response^105^. An open-label, randomized clinical trial showed that C-reactive protein (CRP) levels at baseline predicted treatment outcome differences of two antidepressants^197^. In depressed patients with low levels of CRP (<1 mg/L), escitalopram was more efficacious than nortriptyline, while nortriptyline was more efficacious in patients with higher CRP levels. A meta-analysis of nonsteroidal anti-inflammatory drugs and cytokine inhibitors suggests that anti-inflammatory treatment, in particular celecoxib, decreases depressive symptoms in individuals with major depression or with clinically significant depressive symptoms^198^. The TNF-α antagonist infliximab reduced symptoms of major depression in individuals with baseline high-sensitivity CRP (hs-CRP) greater than 5 mg/L, while individuals with lower hs-CRP concentrations did better with placebo^199^. A review of meta-analyses suggests that nonsteroidal anti-inflammatory drugs (NSAIDs), omega-3 fatty acids, and cytokine antagonists have antidepressant properties in the subgroup of individuals with major depression with evidence of increased inflammatory biomarkers^200^.

## Conclusion

LLD has less favorable response to antidepressants than depression of younger adults, in part because large subgroups (i.e., depression-executive dysfunction syndrome, vascular depression) have a poor response to these agents. Hypotheses have been advanced on the relationships of etiological factors, predisposing factors, and stress with the mechanisms mediating the clinical expression of LLD. Studies testing various aspects of these hypotheses clarified some of the mechanisms of LLD and provided targets for much needed novel treatments.

Targeting modifiable risk factors of vascular disease in mid-life is a logical approach to prevention of vascular depression. Life-style changes and treatment for hypertension and hypercholesterolemia can improve vascular health. Focused studies need to clarify if such agents can prevent LLD or improve response to antidepressants, and which agents are the most efficacious.

The depression-executive dysfunction and the vascular depression syndromes have clinical presentations and neuroimaging findings consistent with frontostriatal abnormalities. Dopamine D2/3 agonists are effective in depression of Parkinson’s disease and their efficacy needs to be assessed in these two syndromes. Computerized cognitive remediation targeting functions of the cognitive control network improved both executive functions and late-life major depression and is a promising approach for future treatment development. Significant progress has been made in neurostimulation treatments for depressed younger adults. TMS targeting deep structures responsible for mood regulation is well tolerated in LLD and its efficacy in syndromes of LLD needs to be studied.

Efficacious psychotherapies for LLD exist but are underutilized in part because of their complexity. Streamlined, stepped psychotherapies targeting depressive symptoms assumed to result from dysfunction of brain networks implicated in LLD can be reasonably easy to learn and may have potential for dissemination. However, their effectiveness needs further investigation.

Depression increases the risk of dementing disorders. While antidepressants are rather ineffective in treating depression of demented patients, earlier long-term use of antidepressants may reduce the risk of future development of dementia. However, confirmation studies are needed.

## References

[1] Han LKM (2018). Epigenetic aging in major depressive disorder. Am. J. Psychiatry.

[2] Penninx BW (2017). Depression and cardiovascular disease: epidemiological evidence on their linking mechanisms. Neurosci. Biobehav. Rev..

[3] Buigues C (2015). The relationship between depression and frailty syndrome: a systematic review. Aging Ment. Health.

[4] Daskalopoulou M (2016). Depression as a risk factor for the initial presentation of twelve cardiac, cerebrovascular, and peripheral arterial diseases: data linkage study of 1.9 million women and men. PLoS ONE.

[5] Pan A, Sun Q, Okereke OI, Rexrode KM, Hu FB (2011). Depression and risk of stroke morbidity and mortality: a meta-analysis and systematic review. JAMA.

[6] Alexopoulos GS (2005). Depression in the elderly. Lancet.

[7] Taylor WD, Aizenstein HJ, Alexopoulos GS (2013). The vascular depression hypothesis: mechanisms linking vascular disease with depression. Mol. Psychiatry.

[8] Alexopoulos GS (2012). Functional connectivity in the cognitive control network and the default mode network in late-life depression. J. Affect. Disord..

[9] Yuen GS (2014). The salience network in the apathy of late-life depression. Int. J. Geriatr. Psychiatry.

[10] Mulders PC, van Eijndhoven PF, Schene AH, Beckmann CF, Tendolkar I (2015). Resting-state functional connectivity in major depressive disorder: a review. Neurosci. Biobehav. Rev..

[11] Manning KJ, Steffens DC (2018). State of the science of neural systems in late-life depression: impact on clinical presentation and treatment outcome. J. Am. Geriatr. Soc..

[12] Alexopoulos GS (2008). Microstructural white matter abnormalities and remission of geriatric depression. Am. J. Psychiatry.

[13] Alexopoulos GS (1997). ‘Vascular depression’ hypothesis. Arch. Gen. Psychiatry.

[14] Januar V, Ancelin ML, Ritchie K, Saffery R, Ryan J (2015). BDNF promoter methylation and genetic variation in late-life depression. Transl. Psychiatry.

[15] Miller AH, Haroon E, Raison CL, Felger JC (2013). Cytokine targets in the brain: impact on neurotransmitters and neurocircuits. Depress. Anxiety.

[16] Hall BS, Moda RN, Liston C (2015). Glucocorticoid mechanisms of functional connectivity changes in stress-related neuropsychiatric disorders. Neurobiol. Stress.

[17] McEwen B (2003). Mood disorders and allostatic load. Biol. Psychiatry.

[18] Diniz BS (2015). Plasma biosignature and brain pathology related to persistent cognitive impairment in late-life depression. Mol. Psychiatry.

[19] Diniz BS (2017). Enhanced molecular aging in late-life depression: the senescent-associated secretory phenotype. Am. J. Geriatr. Psychiatry.

[20] Alexopoulos GS (2001). “The depression-executive dysfunction syndrome of late life”: a specific target for D3 agonists?. Am. J. Geriatr. Psychiatry.

[21] Lim J (2013). Sensitivity of cognitive tests in four cognitive domains in discriminating MDD patients from healthy controls: a meta-analysis. Int. Psychogeriatr..

[22] Alexopoulos GS, Kiosses DN, Klimstra S, Kalayam B, Bruce ML (2002). Clinical presentation of the “depression-executive dysfunction syndrome” of late life. Am. J. Geriatr. Psychiatry.

[23] Alexopoulos GS (1997). Clinically defined vascular depression. Am. J. psychiatry.

[24] Rapp MA (2005). Neuropsychological differences between late-onset and recurrent geriatric major depression. Am. J. Psychiatry.

[25] Taylor WD (2003). Localization of age-associated white matter hyperintensities in late-life depression. Prog. Neuropsychopharmacol. Biol. Psychiatry.

[26] Alexopoulos GS (2009). Serotonin transporter polymorphisms, microstructural white matter abnormalities and remission of geriatric depression. J. Affect. Disord..

[27] Bae JN (2006). Dorsolateral prefrontal cortex and anterior cingulate cortex white matter alterations in late-life depression. Biol. Psychiatry.

[28] Aizenstein HJ (2009). Altered functioning of the executive control circuit in late-life depression: episodic and persistent phenomena. Am. J. Geriatr. Psychiatry.

[29] Yuen GS (2015). Apathy in late-life depression: common, persistent, and disabling. Am. J. Geriatr. Psychiatry.

[30] Hollocks MJ (2015). Differential relationships between apathy and depression with white matter microstructural changes and functional outcomes. Brain.

[31] Alexopoulos GS (2013). Functional connectivity in apathy of late-life depression: a preliminary study. J. Affect. Disord..

[32] Kalayam B, Alexopoulos GS (1999). Prefrontal dysfunction and treatment response in geriatric depression. Arch. Gen. Psychiatry.

[33] Alexopoulos GS (2000). Executive dysfunction and long-term outcomes of geriatric depression. Arch. Gen. Psychiatry.

[34] Sneed JR (2007). Response inhibition predicts poor antidepressant treatment response in very old depressed patients. Am. J. Geriatr. Psychiatry.

[35] Alexopoulos GS, Kiosses DN, Murphy C, Heo M (2004). Executive dysfunction, heart disease burden, and remission of geriatric depression. Neuropsychopharmacology.

[36] Potter GG, Kittinger JD, Wagner HR, Steffens DC, Krishnan KR (2004). Prefrontal neuropsychological predictors of treatment remission in late-life depression. Neuropsychopharmacology.

[37] Sheline YI (2010). Support for the vascular depression hypothesis in late-life depression: results of a 2-site, prospective, antidepressant treatment trial. Arch. Gen. Psychiatry.

[38] Manning KJ (2015). Executive functioning complaints and escitalopram treatment response in late-life depression. Am. J. Geriatr. Psychiatry.

[39] Gunning-Dixon FM (2010). MRI signal hyperintensities and treatment remission of geriatric depression. J. Affect. Disord..

[40] Thompson DG (2015). FMRI activation during executive function predicts response to cognitive behavioral therapy in older, depressed adults. Am. J. Geriatr. Psychiatry.

[41] Krishnan M, Mast BT, Ficker LJ, Lawhorne L, Lichtenberg PA (2005). The effects of preexisting depression on cerebrovascular health outcomes in geriatric continuing care. J. Gerontol. Ser. A.

[42] Williamson W (2018). Association of cardiovascular risk factors with mri indices of cerebrovascular structure and function and white matter hyperintensities in young adults. JAMA.

[43] Arvanitakis Z (2018). Late-life blood pressure association with cerebrovascular and Alzheimer disease pathology. Neurology.

[44] de la Torre JC (2012). Cerebral hemodynamics and vascular risk factors: setting the stage for Alzheimer’s disease. J. Alzheimers Dis..

[45] Paranthaman R (2012). Relationship of endothelial function and atherosclerosis to treatment response in late-life depression. Int. J. Geriatr. Psychiatry.

[46] Rensma SP, van Sloten TT, Launer LJ, Stehouwer CDA (2018). Cerebral small vessel disease and risk of incident stroke, dementia and depression, and all-cause mortality: a systematic review and meta-analysis. Neurosci. Biobehav. Rev..

[47] Steffens DC, Krishnan KR, Crump C, Burke GL (2002). Cerebrovascular disease and evolution of depressive symptoms in the cardiovascular health study. Stroke.

[48] van Sloten TT (2016). Associations between arterial stiffness, depressive symptoms and cerebral small vessel disease: cross-sectional findings from the AGES-Reykjavik Study. J. Psychiatry Neurosci..

[49] van Sloten TT (2015). Cerebral small vessel disease and association with higher incidence of depressive symptoms in a general elderly population: the AGES-Reykjavik study. Am. J. Psychiatry.

[50] Prugger C (2015). Longitudinal association of carotid plaque presence and intima-media thickness with depressive symptoms in the elderly: the three-city study. Arterioscler. Thromb. Vasc. Biol..

[51] Glassman AH, Bigger JT, Gaffney M, Shapiro PA, Swenson JR (2006). Onset of major depression associated with acute coronary syndromes: relationship of onset, major depressive disorder history, and episode severity to sertraline benefit. Arch. Gen. Psychiatry.

[52] Surtees PG (2008). Major depression, C-reactive protein, and incident ischemic heart disease in healthy men and women. Psychosom. Med..

[53] Miller AH, Maletic V, Raison CL (2009). Inflammation and its discontents: the role of cytokines in the pathophysiology of major depression. Biol. Psychiatry.

[54] Zill P (2012). DNA methylation analysis of the angiotensin converting enzyme (ACE) gene in major depression. PLoS One.

[55] Ancelin ML (2013). Angiotensin-converting enzyme gene variants are associated with both cortisol secretion and late-life depression. Transl. Psychiatry.

[56] Tenev VT, Robinson RG, Jorge RE (2009). Is family history of depression a risk factor for poststroke depression? Meta-analysis. Am. J. Geriatr. Psychiatry.

[57] Krishnan KR, Hays JC, Blazer DG (1997). MRI-defined vascular depression. Am. J. Psychiatry.

[58] Reppermund S (2014). White matter integrity and late-life depression in community-dwelling individuals: diffusion tensor imaging study using tract-based spatial statistics. Br. J. Psychiatry..

[59] Taylor WD (2013). Fiber tract-specific white matter lesion severity Findings in late-life depression and by AGTR1 A1166C genotype. Hum. Brain Mapp..

[60] Sheline YI (2008). Regional white matter hyperintensity burden in automated segmentation distinguishes late-life depressed subjects from comparison subjects matched for vascular risk factors. Am. J. Psychiatry.

[61] O’Brien JT (2006). White matter hyperintensities rather than lacunar infarcts are associated with depressive symptoms in older people: the LADIS study. Am. J. Geriatr. Psychiatry.

[62] Wen MC, Steffens DC, Chen MK, Zainal NH (2014). Diffusion tensor imaging studies in late-life depression: systematic review and meta-analysis. Int. J. Geriatr. Psychiatry.

[63] Wu M (2011). Default-mode network connectivity and white matter burden in late-life depression. Psychiatry Res..

[64] Aizenstein HJ (2011). fMRI correlates of white matter hyperintensities in late-life depression. Am. J. Psychiatry.

[65] Santos M (2010). Neuropathological analysis of lacunes and microvascular lesions in late-onset depression. Neuropathol. Appl. Neurobiol..

[66] Tsopelas C (2011). Neuropathological correlates of late-life depression in older people. Br. J. Psychiatry..

[67] Wilson RS (2014). Clinical-pathologic study of depressive symptoms and cognitive decline in old age. Neurology.

[68] Wilson RS (2016). Late-life depression is not associated with dementia-related pathology. Neuropsychology.

[69] Venkatraman VK (2010). Executive control function, brain activation and white matter hyperintensities in older adults. Neuroimage.

[70] Mayda AB, Westphal A, Carter CS, DeCarli C (2011). Late life cognitive control deficits are accentuated by white matter disease burden. Brain.

[71] Sexton CE (2012). Magnetic resonance imaging in late-life depression: vascular and glucocorticoid cascade hypotheses. Br. J. Psychiatry.

[72] Taylor WD, MacFall JR, Gerig G, Krishnan RR (2007). Structural integrity of the uncinate fasciculus in geriatric depression: Relationship with age of onset. Neuropsychiatr. Dis. Treat..

[73] Steffens DC, Taylor WD, Denny KL, Bergman SR, Wang L (2011). Structural integrity of the uncinate fasciculus and resting state functional connectivity of the ventral prefrontal cortex in late life depression. PLoS ONE.

[74] Abi Zeid Daou M, Boyd BD, Donahue MJ, Albert K, Taylor WD (2018). Anterior-posterior gradient differences in lobar and cingulate cortex cerebral blood flow in late-life depression. J. Psychiatr. Res..

[75] Direk N (2012). Cerebral hemodynamics and incident depression: the Rotterdam study. Biol. Psychiatry.

[76] Brickman AM (2009). Reduction in cerebral blood flow in areas appearing as white matter hyperintensities on magnetic resonance imaging. Psychiatry Res..

[77] van Sloten TT (2014). Endothelial dysfunction is associated with a greater depressive symptom score in a general elderly population: the Hoorn Study. Psychol. Med..

[78] Liao W (2017). Cerebral blood flow changes in remitted early- and late-onset depression patients. Oncotarget.

[79] van Agtmaal MJM, Houben A, Pouwer F, Stehouwer CDA, Schram MT (2017). Association of microvascular dysfunction with late-life depression: a systematic review and meta-analysis. JAMA Psychiatry.

[80] Smagula SF (2017). Immunological biomarkers associated with brain structure and executive function in late-life depression: exploratory pilot study. Int. J. Geriatr. Psychiatry.

[81] Almeida OP (2008). Homocysteine and depression in later life. Arch. Gen. Psychiatry.

[82] Satizabal CL, Zhu YC, Mazoyer B, Dufouil C, Tzourio C (2012). Circulating IL-6 and CRP are associated with MRI findings in the elderly: the 3C-Dijon Study. Neurology.

[83] Dorr A (2012). Amyloid-beta-dependent compromise of microvascular structure and function in a model of Alzheimer’s disease. Brain.

[84] Alexopoulos GS, Morimoto SS (2011). The inflammation hypothesis in geriatric depression. Int. J. Geriatr. Psychiatry.

[85] Gruver AL, Hudson LL, Sempowski GD (2007). Immunosenescence of ageing. J. Pathol..

[86] Dilger RN, Johnson RW (2008). Aging, microglial cell priming, and the discordant central inflammatory response to signals from the peripheral immune system. J. Leukoc. Biol..

[87] Lucin KM, Wyss-Coray T (2009). Immune activation in brain aging and neurodegeneration: too much or too little?. Neuron.

[88] Dantzer R, O’Connor JC, Lawson MA, Kelley KW (2011). Inflammation-associated depression: from serotonin to kynurenine. Psychoneuroendocrinology.

[89] Haroon E (2016). Conceptual convergence: increased inflammation is associated with increased basal ganglia glutamate in patients with major depression. Mol. Psychiatry.

[90] Haroon E (2014). IFN-alpha-induced cortical and subcortical glutamate changes assessed by magnetic resonance spectroscopy. Neuropsychopharmacology.

[91] Haroon E (2015). Age-related increases in basal ganglia glutamate are associated with TNF, reduced motivation and decreased psychomotor speed during IFN-alpha treatment: preliminary findings. Brain Behav. Immun..

[92] Kiecolt-Glaser JK, Derry HM, Fagundes CP (2015). Inflammation: depression fans the flames and feasts on the heat. Am. J. Psychiatry.

[93] Slavich GM, Irwin MR (2014). From stress to inflammation and major depressive disorder: a social signal transduction theory of depression. Psychol. Bull..

[94] Setiawan E (2018). Association of translocator protein total distribution volume with duration of untreated major depressive disorder: a cross-sectional study. Lancet Psychiatry.

[95] Thomas AJ, Ferrier IN, Kalaria RN, Davis S, O’Brien JT (2002). Cell adhesion molecule expression in the dorsolateral prefrontal cortex and anterior cingulate cortex in major depression in the elderly. Br. J. Psychiatry..

[96] Baune Bernhard T., Dannlowski Udo, Domschke Katharina, Janssen Debbie G.A., Jordan Margaret A., Ohrmann Patricia, Bauer Jochen, Biros Erik, Arolt Volker, Kugel Harald, Baxter Alan G., Suslow Thomas (2010). The Interleukin 1 Beta (IL1B) Gene Is Associated with Failure to Achieve Remission and Impaired Emotion Processing in Major Depression. Biological Psychiatry.

[97] Capuron L (2005). Anterior cingulate activation and error processing during interferon-alpha treatment. Biol. Psychiatry.

[98] Alexopoulos GS, Gunning-Dixon FM, Latoussakis V, Kanellopoulos D, Murphy CF (2008). Anterior cingulate dysfunction in geriatric depression. Int. J. Geriatr. Psychiatry.

[99] Harrison NA (2009). Inflammation causes mood changes through alterations in subgenual cingulate activity and mesolimbic connectivity. Biol. Psychiatry.

[100] Gaarden TL (2018). Exploration of 27 plasma immune markers: a cross-sectional comparison of 64 old psychiatric inpatients having unipolar major depression and 18 non-depressed old persons. BMC Geriatr..

[101] Gimeno D (2009). Associations of C-reactive protein and interleukin-6 with cognitive symptoms of depression: 12-year follow-up of the Whitehall II study. Psychol. Med..

[102] Kohler CA (2017). Peripheral cytokine and chemokine alterations in depression: a meta-analysis of 82 studies. Acta Psychiatr. Scand..

[103] Lindqvist D (2009). Interleukin-6 is elevated in the cerebrospinal fluid of suicide attempters and related to symptom severity. Biol. Psychiatry.

[104] Milaneschi Y (2009). Interleukin-1 receptor antagonist and incident depressive symptoms over 6 years in older persons: the InCHIANTI study. Biol. Psychiatry.

[105] Kohler CA (2018). Peripheral alterations in cytokine and chemokine levels after antidepressant drug treatment for major depressive disorder: systematic review and meta-analysis. Mol. Neurobiol..

[106] Apostolakis S, Vogiatzi K, Krambovitis E, Spandidos DA (2008). IL-1 cytokines in cardiovascular disease: diagnostic, prognostic and therapeutic implications. Cardiovasc Hematol. Agents Med. Chem..

[107] Sparkman NL, Johnson RW (2008). Neuroinflammation associated with aging sensitizes the brain to the effects of infection or stress. Neuroimmunomodulation.

[108] Irwin MR, Cole SW (2011). Reciprocal regulation of the neural and innate immune systems. Nat. Rev. Immunol..

[109] Mahgoub N, Alexopoulos GS (2016). The amyloid hypothesis: is there a role for anti-amyloid treatment in late-life depression?. Am. J. Geriatr. Psychiatry..

[110] Yasuno F (2016). High amyloid-beta deposition related to depressive symptoms in older individuals with normal cognition: a pilot study. Int. J. Geriatr. Psychiatry.

[111] Donovan NJ (2018). Longitudinal association of amyloid beta and anxious-depressive symptoms in cognitively normal older adults. Am. J. Psychiatry.

[112] Wu KY (2014). Increased brain amyloid deposition in patients with a lifetime history of major depression: evidenced on 18F-florbetapir (AV-45/Amyvid) positron emission tomography. Eur. J. Nucl. Med. Mol. Imaging.

[113] Gallagher D, Kiss A, Lanctot K, Herrmann N (2018). Depression and risk of alzheimer dementia: a longitudinal analysis to determine predictors of increased risk among older adults with depression. Am. J. Geriatr. Psychiatry.

[114] Chung JK (2015). Lifetime history of depression predicts increased amyloid-beta accumulation in patients with mild cognitive impairment. J. Alzheimers Dis..

[115] Rapp MA (2006). Increased hippocampal plaques and tangles in patients with Alzheimer disease with a lifetime history of major depression. Arch. Gen. Psychiatry.

[116] Sun X (2008). Amyloid-associated depression: a prodromal depression of Alzheimer disease?. Arch. Gen. Psychiatry.

[117] Sheline YI (2014). An antidepressant decreases CSF Abeta production in healthy individuals and in transgenic AD mice. Sci. Transl. Med..

[118] Cirrito JR (2011). Serotonin signaling is associated with lower amyloid-beta levels and plaques in transgenic mice and humans. Proc. Natl Acad. Sci. USA.

[119] Bartels C, Wagner M, Wolfsgruber S, Ehrenreich H, Schneider A (2018). Impact of SSRI therapy on risk of conversion from mild cognitive impairment to Alzheimer’s dementia in individuals with previous depression. Am. J. Psychiatry.

[120] De Winter FL (2017). No association of lower hippocampal volume with Alzheimer’s disease pathology in late-life depression. Am. J. Psychiatry.

[121] Madsen K (2012). Lack of association between prior depressive episodes and cerebral [11C]PiB binding. Neurobiol. Aging.

[122] O’Brien J (2001). Cognitive impairment in depression is not associated with neuropathologic evidence of increased vascular or Alzheimer-type pathology. Biol. Psychiatry.

[123] Hendricksen M, Thomas AJ, Ferrier IN, Ince P, O’Brien JT (2004). Neuropathological study of the dorsal raphe nuclei in late-life depression and Alzheimer’s disease with and without depression. Am. J. Psychiatry.

[124] McCutcheon ST (2016). Clinicopathological correlates of depression in early Alzheimer’s disease in the NACC. Int. J. Geriatr. Psychiatry.

[125] Brown EE, Iwata Y, Chung JK, Gerretsen P, Graff-Guerrero A (2016). Tau in late-life depression: a systematic review and meta-analysis. J. Alzheimers Dis..

[126] Schaakxs R (2018). Associations between age and the course of major depressive disorder: a 2-year longitudinal cohort study. Lancet Psychiatry.

[127] Cipriani A (2018). Comparative efficacy and acceptability of 21 antidepressant drugs for the acute treatment of adults with major depressive disorder: a systematic review and network meta-analysis. Lancet.

[128] Tedeschini E (2011). Efficacy of antidepressants for late-life depression: a meta-analysis and meta-regression of placebo-controlled randomized trials. J. Clin. Psychiatry.

[129] Kok RM, Nolen WA, Heeren TJ (2012). Efficacy of treatment in older depressed patients: a systematic review and meta-analysis of double-blind randomized controlled trials with antidepressants. J. Affect. Disord..

[130] Kok RM, Reynolds CF (2017). Management of depression in older adults: a review. JAMA.

[131] Cooper C (2011). A systematic review of treatments for refractory depression in older people. Am. J. Psychiatry.

[132] Lenze EJ (2015). Efficacy, safety, and tolerability of augmentation pharmacotherapy with aripiprazole for treatment-resistant depression in late life: a randomised, double-blind, placebo-controlled trial. Lancet.

[133] Lavretsky H (2015). Citalopram, methylphenidate, or their combination in geriatric depression: a randomized, double-blind, placebo-controlled trial. Am. J. Psychiatry.

[134] Reynolds CF (2011). Maintenance treatment of depression in old age: a randomized, double-blind, placebo-controlled evaluation of the efficacy and safety of donepezil combined with antidepressant pharmacotherapy. Arch. Gen. Psychiatry.

[135] Devanand DP (2018). Donepezil treatment in patients with depression and cognitive impairment on stable antidepressant treatment: a randomized controlled trial. Am. J. Geriatr. Psychiatry.

[136] Mitchell AJ, Subramaniam H (2005). Prognosis of depression in old age compared to middle age: a systematic review of comparative studies. Am. J. Psychiatry.

[137] Kok RM, Heeren TJ, Nolen WA (2011). Continuing treatment of depression in the elderly: a systematic review and meta-analysis of double-blinded randomized controlled trials with antidepressants. Am. J. Geriatr. Psychiatry.

[138] Reynolds CF (2006). Maintenance treatment of major depression in old age. New Engl. J. Med..

[139] Reynolds CF (1999). Nortriptyline and interpersonal psychotherapy as maintenance therapies for recurrent major depression: a randomized controlled trial in patients older than 59 years. JAMA.

[140] Borges S (2014). Review of maintenance trials for major depressive disorder: a 25-year perspective from the US Food and Drug Administration. J. Clin. Psychiatry.

[141] Deng Y (2018). Predictors of recurrence in remitted late-life depression. Depress. Anxiety.

[142] Alexopoulos, G. S., Katz, I. R., Reynolds, C. F., 3rd, Carpenter, D., Docherty, J. P. The expert consensus guideline series. Pharmacotherapy of depressive disorders in older patients. *Postgraduate Med.* **110**, 1–86 (2001). 17205639

[143] Kiosses DN, Alexopoulos GS (2013). The prognostic significance of subsyndromal symptoms emerging after remission of late-life depression. Psychol. Med..

[144] Hedayati SS (2017). Effect of sertraline on depressive symptoms in patients with chronic kidney disease without dialysis dependence: the CAST randomized clinical trial. JAMA.

[145] Mulick A (2018). Does depression treatment improve the survival of depressed patients with cancer? A long-term follow-up of participants in the SMaRT Oncology-2 and 3 trials. Lancet Psychiatry.

[146] Pizzi C (2011). Meta-analysis of selective serotonin reuptake inhibitors in patients with depression and coronary heart disease. Am. J. Cardiol..

[147] Glassman AH (2002). Sertraline treatment of major depression in patients with acute MI or unstable angina. JAMA.

[148] Honig A (2007). Treatment of post-myocardial infarction depressive disorder: a randomized, placebo-controlled trial with mirtazapine. Psychosom. Med..

[149] Lesperance F (2007). Effects of citalopram and interpersonal psychotherapy on depression in patients with coronary artery disease: the Canadian Cardiac Randomized Evaluation of Antidepressant and Psychotherapy Efficacy (CREATE) trial. JAMA.

[150] Kim JM (2018). Effect of escitalopram vs placebo treatment for depression on long-term cardiac outcomes in patients with acute coronary syndrome: a randomized clinical trial. JAMAama.

[151] Nelson JC, Devanand DP (2011). A systematic review and meta-analysis of placebo-controlled antidepressant studies in people with depression and dementia. J. Am. Geriatr. Soc..

[152] Kessing LV, Sondergard L, Forman JL, Andersen PK (2008). Lithium treatment and risk of dementia. Arch. Gen. Psychiatry.

[153] Zivin K, Kales HC (2008). Adherence to depression treatment in older adults: a narrative review. Drugs Aging.

[154] Spaans HP (2015). Speed of remission in elderly patients with depression: electroconvulsive therapy v. medication. Br. J. Psychiatry.

[155] Taylor WD (2014). Clinical practice. Depression in the elderly. New Engl. J. Med..

[156] Slade EP, Jahn DR, Regenold WT, Case BG (2017). Association of electroconvulsive therapy with psychiatric readmissions in US hospitals. JAMA psychiatry.

[157] Kerner N, Prudic J (2014). Current electroconvulsive therapy practice and research in the geriatric population. Neuropsychiatry.

[158] Tor PC (2015). A systematic review and meta-analysis of brief versus ultrabrief right unilateral electroconvulsive therapy for depression. J. Clin. Psychiatry.

[159] Kellner CH (2016). Right unilateral ultrabrief pulse ECT in geriatric depression: phase 1 of the PRIDE study. Am. J. Psychiatry.

[160] Kiosses DN, Leon AC, Arean PA (2011). Psychosocial interventions for late-life major depression: evidence-based treatments, predictors of treatment outcomes, and moderators of treatment effects. Psychiatr. Clin. North Am..

[161] Pinquart M, Duberstein PR, Lyness JM (2006). Treatments for later-life depressive conditions: a meta-analytic comparison of pharmacotherapy and psychotherapy. Am. J. Psychiatry.

[162] Arean PA (2010). Problem-solving therapy and supportive therapy in older adults with major depression and executive dysfunction. Am. J. Psychiatry.

[163] Alexopoulos GS (2011). Problem-solving therapy and supportive therapy in older adults with major depression and executive dysfunction: effect on disability. Arch. Gen. Psychiatry.

[164] Bella R (2010). Clinical presentation and outcome of geriatric depression in subcortical ischemic vascular disease. Gerontology.

[165] Alexopoulos GS (2016). Two behavioral interventions for patients with major depression and severe COPD. Am. J. Geriatr. Psychiatry.

[166] Alexopoulos GS (2018). Two interventions for patients with major depression and severe chronic obstructive pulmonary disease: impact on dyspnea-related disability. Am. J. Geriatr. Psychiatry.

[167] Alexopoulos GS (2013). Personalised intervention for people with depression and severe COPD. Br. J. Psychiatry.

[168] Alexopoulos GS (2016). Clinical case management versus case management with problem-solving therapy in low-income, disabled elders with major depression: a randomized clinical trial. Am. J. Geriatr. Psychiatry.

[169] NAS. (2015). Standrards for Psychosocial Interventions..

[170] Alexopoulos GS, Arean P (2014). A model for streamlining psychotherapy in the RDoC era: the example of ‘Engage’. Mol. Psychiatry.

[171] Alexopoulos GS (2015). Comparing engage with PST in late-life major depression: a preliminary report. Am. J. Geriatr. Psychiatry.

[172] Alexopoulos GS (2016). “Engage” therapy: behavioral activation and improvement of late-life major depression. Am. J. Geriatr. Psychiatry.

[173] Alexopoulos GS (2017). “Engage” therapy: prediction of change of late-life major depression. J. Affect. Disord..

[174] Willner P, Hale AS, Argyropoulos S (2005). Dopaminergic mechanism of antidepressant action in depressed patients. J. Affect. Disord..

[175] Barone P (2010). Pramipexole for the treatment of depressive symptoms in patients with Parkinson’s disease: a randomised, double-blind, placebo-controlled trial. Lancet Neurol..

[176] Inoue T (2010). Pramipexole for stage 2 treatment-resistant major depression: an open study. Prog. Neuropsychopharmacol. Biol. Psychiatry.

[177] Hori H, Kunugi H (2012). The efficacy of pramipexole, a dopamine receptor agonist, as an adjunctive treatment in treatment-resistant depression: an open-label trial. Sci. World J..

[178] Corrigan MH, Denahan AQ, Wright CE, Ragual RJ, Evans DL (2000). Comparison of pramipexole, fluoxetine, and placebo in patients with major depression. Depress. Anxiety.

[179] Anguera JA, Gunning FM, Arean PA (2017). Improving late life depression and cognitive control through the use of therapeutic video game technology: A proof-of-concept randomized trial. Depress. Anxiety.

[180] Morimoto SS (2014). Neuroplasticity-based computerized cognitive remediation for treatment-resistant geriatric depression. Nat. Commun..

[181] Dubin MJ, Liston C, Avissar MA, Ilieva I, Gunning FM (2017). Network-guided transcranial magnetic stimulation for depression. Curr. Behav. Neurosci. Rep..

[182] Kaster TS (2018). Efficacy, tolerability, and cognitive effects of deep transcranial magnetic stimulation for late-life depression: a prospective randomized controlled trial. Neuropsychopharmacology.

[183] Jorge RE, Moser DJ, Acion L, Robinson RG (2008). Treatment of vascular depression using repetitive transcranial magnetic stimulation. Arch. Gen. Psychiatry.

[184] Muntner P (2018). Potential U.S. population impact of the 2017 ACC/AHA high blood pressure guideline. J. Am. Coll. Cardiol..

[185] Dupuis F, Atkinson J, Liminana P, Chillon JM (2005). Captopril improves cerebrovascular structure and function in old hypertensive rats. Br. J. Pharmacol..

[186] Morimoto S, Maki K, Aota Y, Sakuma T, Iwasaka T (2008). Beneficial effects of combination therapy with angiotensin II receptor blocker and angiotensin-converting enzyme inhibitor on vascular endothelial function. Hypertens. Res..

[187] Hajjar I (2012). Effect of antihypertensive therapy on cognitive function in early executive cognitive impairment: a double-blind randomized clinical trial. Arch. Intern. Med..

[188] Moriwaki H (2004). Losartan, an angiotensin II (AT1) receptor antagonist, preserves cerebral blood flow in hypertensive patients with a history of stroke. J. Hum. Hypertens..

[189] Wilms H, Rosenstiel P, Unger T, Deuschl G, Lucius R (2005). Neuroprotection with angiotensin receptor antagonists: a review of the evidence and potential mechanisms. Am. J. Cardiovasc. Drug..

[190] Nagata R, Kawabe K, Ikeda K (2010). Olmesartan, an angiotensin II receptor blocker, restores cerebral hypoperfusion in elderly patients with hypertension. J. Stroke Cereb. Dis..

[191] Pavlatou MG (2008). Chronic administration of an angiotensin II receptor antagonist resets the hypothalamic-pituitary-adrenal (HPA) axis and improves the affect of patients with diabetes mellitus type 2: preliminary results. Stress.

[192] Taragano FE, Allegri R, Vicario A, Bagnatti P, Lyketsos CG (2001). A double blind, randomized clinical trial assessing the efficacy and safety of augmenting standard antidepressant therapy with nimodipine in the treatment of ‘vascular depression’. Int. J. Geriatr. Psychiatry.

[193] Taragano FE, Bagnatti P, Allegri RF (2005). A double-blind, randomized clinical trial to assess the augmentation with nimodipine of antidepressant therapy in the treatment of “vascular depression”. Int. Psychogeriatr..

[194] Ried LD, Tueth MJ, Handberg E, Kupfer S, Pepine CJ (2005). A Study of Antihypertensive Drugs and Depressive Symptoms (SADD-Sx) in patients treated with a calcium antagonist versus an atenolol hypertension Treatment Strategy in the International Verapamil SR-Trandolapril Study (INVEST). Psychosom. Med..

[195] Parsaik AK (2014). Statins use and risk of depression: a systematic review and meta-analysis. J. Affect. Disord..

[196] Vogelzangs N (2014). Inflammatory and metabolic dysregulation and the 2-year course of depressive disorders in antidepressant users. Neuropsychopharmacology.

[197] Uher R (2014). An inflammatory biomarker as a differential predictor of outcome of depression treatment with escitalopram and nortriptyline. Am. J. Psychiatry.

[198] Kohler O (2014). Effect of anti-inflammatory treatment on depression, depressive symptoms, and adverse effects: a systematic review and meta-analysis of randomized clinical trials. JAMA Psychiatry.

[199] Raison CL (2013). A randomized controlled trial of the tumor necrosis factor antagonist infliximab for treatment-resistant depression: the role of baseline inflammatory biomarkers. JAMA Psychiatry.

[200] Raison CL (2017). The promise and limitations of anti-inflammatory agents for the treatment of major depressive disorder. Curr. Top. Behav. Neurosci..

